# Prognostic Role of CD68^+^ and CD163^+^ Tumour-Associated Macrophages and PD-L1 Expression in Oral Squamous Cell Carcinoma: A Meta-Analysis

**DOI:** 10.3389/bjbs.2023.11065

**Published:** 2023-06-16

**Authors:** Mohammed Haseeb Chohan, Matthew Perry, Paul Laurance-Young, Vehid M. Salih, Andrew D. Foey

**Affiliations:** ^1^ School of Biomedical Sciences, Faculty of Health, University of Plymouth, Plymouth, United Kingdom; ^2^ School of Dentistry, Faculty of Health, University of Plymouth, Plymouth, United Kingdom

**Keywords:** macrophages, oral cancer, CD68, PD-L1, CD163

## Abstract

**Background:** Oral squamous cell carcinoma (OSCC) is a common malignant cancer in humans. An abundance of tumour associated macrophages (TAMs) create an immunosuppressive tumour microenvironment (TME). TAM markers (CD163 and CD68) are seen to serve as prognostic factors in OSCC. PD-L1 has seen to widely modulate the TME but its prognostic significance remains controversial. The aim of this meta-analysis is to evaluate the prognostic role of CD163^+^, CD68^+^ TAMs and PD-L1 in OSCC patients.

**Methods:** Searches in PubMed, Scopus and Web of Science were performed; 12 studies were included in this meta-analysis. Quality assessment of included studies was performed according to REMARK guidelines. Risk of bias across studies was investigated according to the rate of heterogeneity. Meta-analysis was performed to investigate the association of all three biomarkers with overall survival (OS).

**Results:** High expression of CD163^+^ TAMs were associated with poor overall survival (HR = 2.64; 95% Cl: [1.65, 4.23]; *p* < 0.0001). Additionally, high stromal expression of CD163^+^ TAMs correlated with poor overall survival (HR = 3.56; 95% Cl: [2.33, 5.44]; *p* < 0.00001). Conversely, high CD68 and PD-L1 expression was not associated with overall survival (HR = 1.26; 95% Cl: [0.76, 2.07]; *p* = 0.37) (HR = 0.64; 95% Cl: [0.35, 1.18]; *p* = 0.15).

**Conclusion:** In conclusion, our findings indicate CD163^+^ can provide prognostic utility in OSCC. However, our data suggests CD68^+^ TAMs were not associated with any prognostic relevance in OSCC patients, whereas PD-L1 expression may prove to be a differential prognostic marker dependent on tumour location and stage of progression.

## Introduction

Oral squamous cell carcinoma (OSCC) is a common malignant neoplasm (80%–90%) of the oral cavity, derived from the head and neck region of the body. It is associated with the common risk factors of smoking and alcohol consumption (1, 2). Contributing the highest incidence and mortality rate in both males and females, there were 354,864 new cases and 177,384 deaths worldwide in 2018 and was the leading cause of mortality in Central Asia (3). Whilst there is an improvement in advancing therapies such as surgery and chemotherapy, the 5-year survival rate remains 50% in various countries over the past 3 decades (4). This insufficient improvement in prognosis could be explained by the lack of consideration of immunological parameters in prognostic classification and treatment of OSCC (5). Moreover, poor prognosis in OSCC may be a result of its aggressive local invasion and metastasis, leading to an uncontrollable recurrence (6).

Metastasis is achieved through the interaction of tumour cells and the surrounding tumour microenvironment (TME) (7). This TME plays a critical role in tumourigenesis, tumour progression, invasion and tumour tissue infiltration by tumour-associated macrophages (TAMs) (8). TAMs are abundant in both the tumour and tumour stroma, playing a significant role in cancer progression (9, 10). MCP-1 (Monocyte chemoattractant protein-1 or CCL2) plays a role in recruiting and attracting TAMs to tumour sites (11, 12). These TAMs may exhibit either of two functional phenotypes, M1 or M2, dependent on cytokine, chemokine, chemokine receptor and other regulator expression (13). M1 TAMs exhibit pro-inflammatory and anti-tumoural properties, mediated by IL-12, TNF-α, IFN-γ, and stimulate strong Th1 IFNγ-driven cell mediated responses resulting in tumouricidal function. M2 TAMs are generally anti-inflammatory and pro-tumoural, expressing IL-10, IL-13, MR (mannose receptor) and are capable of inducing humoral Th2-driven cytokine responses, secreting IL-4, IL-13 and high levels of chemokines and growth factors such as VEGF, TGF-β, FGF and uPA, promoting angiogenesis, immunosuppression, tumour invasion and metastasis (14, 15).

TAM polarisation to distinct M1 and M2 subsets however, remains unclear, as recent evidence suggests functional plasticity and the ability to repolarise from one phenotype to the other (16). Human ovarian cancer TAMs have been observed to repolarise from M2 to M1-like phenotype suppressing levels of CCL18, MMP9 and VEGF when exposed to IFN-γ (17). TME TAMs have been shown to favour tumourigenesis, tumour survival and angiogenesis (18). This role in the TME however, is controversial; in colorectal cancer, for example, TAMs exhibit pro-inflammatory anti-tumour effects, leading to a favourable prognosis (19, 20). This may be explained by these M1 TAMs inducing the secretion of galectin-3 in human colon cells which further induces TAM infiltration and release of the pro-inflammatory cytokines, IL-1β and TNF-α, causing a strong anti-tumour response (20). Nevertheless, whilst TAMs may exhibit either phenotype, studies recognise TAMs to be predominantly of the M2 phenotype and correlate with a poor prognosis (15, 21).

TAMs may thus serve as potential biomarkers for the prognosis and therapeutic targeting of several cancers, particularly OSCC. Interestingly, over 80% of studies reveal a high number of TAMs correlates with poor patient prognosis (21). Investigations have shown CD163 (M2 macrophage class B scavenger receptor) (22), as a biomarker for macrophage activation in lung, breast and hepatocellular carcinoma (23-25). Recently, overexpression of M2-like CD163^+^ TAMs in head and neck squamous cell carcinoma (HNSCC) patients, revealed a poor clinical prognosis in both overall survival (OS) and progression-free survival (PFS) (26). CD163 functionality involves eradicating and endocytosing the haemoglobin/haptoglobin complex, thereby protecting tissues from oxidative damage (27). Used as a biomarker for M2c deactivated macrophages, it presents both anti-inflammatory and pro-tumoural functions (28).

Monocyte/macrophages, specifically M2 macrophages, abundantly express CD68, a glycosylated type I transmembrane glycoprotein belonging to the LAMP (lysosomal-associated membrane proteins) family (29). Its primary function is poorly understood, but as a class D scavenger receptor, it plays a role in promoting phagocytosis, clearing cellular debris and mediates recruitment/activation of macrophages (30). In contrast, CD68 is considered to be a pan-macrophage marker expressed by both M1 and M2 subsets, derived from anti-CD14-purified peripheral blood monocytes (31). This may explain observations where overexpression of CD68^+^ TAMs was associated with poor overall survival and disease-free survival (DFS) in breast cancer patients (32), whereas conversely, high CD68^+^ TAM expression conferred a longer overall survival and disease-free survival in hepatocellular carcinoma patients (33). Therefore, its prognostic relevance to OSCC needs clarification.

PD-L1 (programmed death ligand-1 also known as CD274, B7-H1) is a cell surface type I glycoprotein expressed on antigen-presenting cells (APCs) and located in dendritic cells and macrophages. Belonging to the B7 family, it is a co-inhibitory ligand which binds PD-1 (programmed death receptor-1). PD-1 functions as a T-cell checkpoint protein, regulating T-cell suppression (34). PD-1 is a member of CTLA-4 (cytotoxic T lymphocyte-associated protein 4) family, primarily expressed by cytotoxic T cells (Tc), which predominate anti-tumour responses. PD-1 ligation suppresses T-cell function *via* an inhibitory signal involving SHP-2 which inhibits CD28-mediated PI3K and Akt activity (35). In various cancers, PD-1 can be expressed on tumour infiltrating lymphocytes (TILs), where CD4^+^ and CD8^+^ TILs exhibit an increased PD-1 expression on Treg and Tc, effectively resulting in Treg-mediated immunosuppression and Tc anergy/loss of CTL function (36). In order to maintain homeostasis, PD-1/PD-L1 induces immune tolerance and effectively suppresses excessive tissue inflammation and autoimmune disease. In tumours, however, binding of PD-L1 to its PD-1 receptor on activated T cells results in T-cell suppression and immune escape by inhibiting perforin/granzyme production, suppressing IL-2 and IFN-γ production and promoting apoptosis, effectively inducing tumour growth (37). Several studies investigated the relationship between TAM PD-L1 expression and cancer patient prognosis. High PD-L1 expression revealed a poor clinical prognosis in malignant pleural mesothelioma (MPM) and renal cell carcinoma patients (38, 39), whereas other studies reached controversial and inconsistent conclusions. Conversely, high PD-L1 expression was associated with longer overall and disease-free survival (40, 41). Therefore, its prognostic relevance needs further clarification.

This study aims to investigate the prognostic role of CD68^+^, CD163^+^ TAMs and PD-L1 expression in OSCC, through a meta-analysis of the current literature. Furthermore, the prognostic role of these biomarkers was investigated in different sub-locations in OSCC (tumour versus stroma). This study hypothesised a high expression of CD68^+^, CD163^+^ TAMs and PD-L1 would lead to worse survival in OSCC patients, whereas concluded that CD163^+^ TAMs located in both tumour and stroma were predictive of a poor prognosis in OSCC and that PD-L1 may prove to be indicative of a positive outcome in these patients.

## Materials and Methods

### Search Strategy

In order to identify potential studies, a systematic search was conducted on the following online databases: PubMed, Scopus and Web of Science. Two Boolean operators (AND, OR) were used to select specific keywords. The following terms include: (macrophage OR TAM OR “tumour-associated macrophage” OR CD68 OR CD163) AND (“oral cancer” OR “oral squamous cell carcinoma” OR OSCC) AND (survival OR prognosis OR mortality OR death) AND (PD-L1 OR programmed death ligand 1 OR PDL1 OR B7-H1). Title and abstracts were screened based on inclusion and exclusion criteria (Refer to eligibility criteria section below). After inspecting full texts, the final predetermined articles were selected.

## Eligibility Criteria—Included and Excluded Studies

Studies that had met the following inclusion criteria were included in the meta-analysis: 1) English language publication. 2) Studies that reported the prognostic significance and role of CD163, CD68 and PD-L1 in OSCC 3) Studies analysing the protein level expression of CD163, CD68 and PD-L1 in clinical analysis such as immunohistochemistry (IHC) sections in OSCC. 4) Evaluate the association of CD163, CD68 and PD-L1 and patient prognosis according to the following parameters: overall survival (OS). 5) Provided sufficient survival data which included only hazard ratio (HR) with 95% confidence interval (Cl), *p*-value *P*) alongside Kaplan Meier survival graphs. Studies that had less than 30 patients and did not meet the parameters were excluded from the meta-analysis.

### Data Extraction (Outcomes)

Included studies that met the criteria had their extracted data in accordance to: name of first author, year of publication, region of study, sample size, age, type of biomarkers used (CD163, CD68 or both and PD-L1), stage of cancer (TNM stage), location of tumour analysed, follow-up, cut-off values (threshold for prognostic factor and corresponding outcome based on high-risk and low-risk groups) and univariate and/or multivariate analysis outcomes to extract HR and 95% Cl for OS. Articles providing survival data are visualised in Kaplan-Meier curves.

### Risk of Bias

To determine the risk of bias for each study, a quality assessment was conducted in accordance with the REMARK (Reporting Recommendations for Tumour Marker Prognostic Studies) (42). The risk of bias consists of six components: 1) samples, 2) clinical data of the group, 3) immunohistochemistry, 4) prognostication, 5) statistics and 6) prognostic factors. Each component was considered as: sufficient, insufficient or N/A (no description). The assessment scores for each study are shown in Table 1 according to REMARK assessment criteria guidelines presented in Supplementary Table S2.

**TABLE 1:** Quality assessment of studies included in the meta-analysis according to REMARK guidelines.

Author/year [References]	Country	Samples	Clinical data	Immunohistochemistry	Prognostication	Statistics	Prognostic factors
Fujii/2012 (44)	Japan	S	S	S	S	S	S
Fujita/2014 (45)	China	S	S	S	I	I	S
Wang/2014 (46)	China	S	S	S	S	S	S
Matsuoka/2015 (47)	Japan	S	S	S	S	S	S
Takahashi/2017 (48)	Japan	S	S	S	S	S	S
Ni/2015 (49)	China	S	S	S	S	S	S
Fang/2017 (50)	China	S	S	S	S	I	S
Kikuchi/2021 (51)	Japan	S	S	S	I	S	S
Lin/2015 (52)	Taiwan	S	S	S	S	I	S
Kogashiwa/2017 (40)	Japan	S	S	S	S	S	S
Ahn/2017 (53)	South Korea	S	S	S	I	S	S
Lenouvel/2021 (54)	Spain	S	S	S	S	I	S

Included studies scaled: S, sufficient; I, insufficient; N/A, no description.

### Quantitative Data Analysis

RevMan (Review Manager) 5.4.1 was used to extract and construct quantitative data for the meta-analysis. Hazard ratios (either univariate or multivariate estimates), 95% confidence intervals (95% Cl) and *p*-value *P*) were extracted from the included studies. This data was constructed in forest plots with all in a random effect model. HRs for all immune biomarkers were sorted in a high vs. low direction. If HR estimates are reported in the opposite direction, HR and 95% Cl values were inverted. An HR >1 corresponds to worse survival in the group with high CD68^+^, CD163^+^ TAMs or PD-L1 expression. Estimated values of CD163, CD68 and PD-L1 expression were performed based on survival variables such as overall survival (OS). Other survival rates including disease-free survival (DFS) and disease-specific survival (DSS) was not included due to insufficient data. *p*-value lower than 0.05 was considered statistically significant. Heterogeneity (I^2^) is assessed and classified by Higgins index with: low heterogeneity (25%), medium heterogeneity (50%) and high heterogeneity (70%) (43).

## Results

### Study Selection and Study Results

**FIGURE 1:**
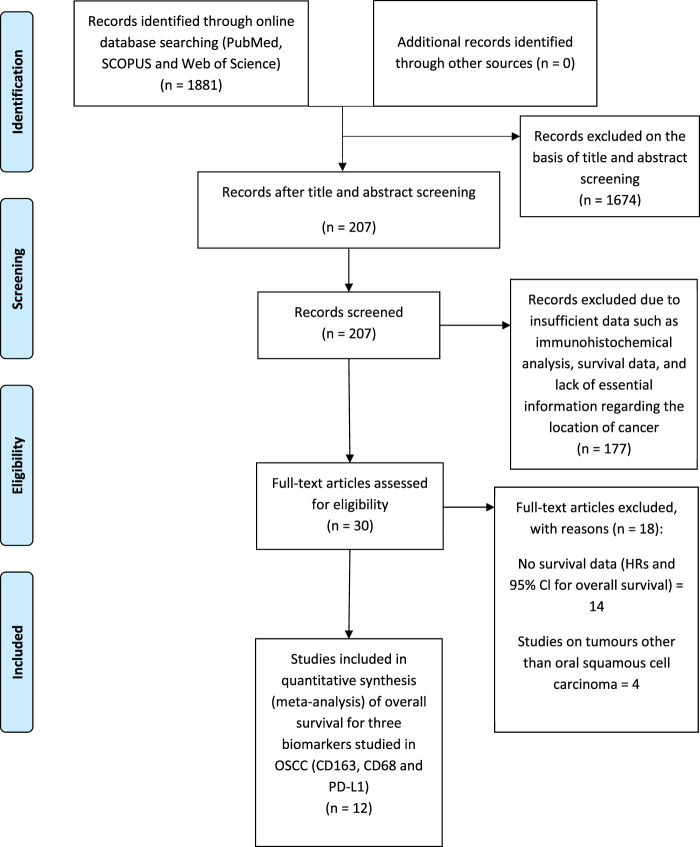
PRISMA (Preferred Reporting Items for Systematic reviews and Meta-Analysis) flow diagram of the study selection process for meta-analysis summarising literature searching, screening and assessment of eligibility of identified studies.

Searches revealed 1881 records from commonly used databases (PubMed, Scopus and Web of Science) which justified the best array of literature. A total of 207 records were screened by title and abstract. Of these 207 articles, 177 were excluded due to providing insufficient data. 30 articles met the initial assessment of the inclusion criteria. Eventually, 12 studies were considered for inclusion in the meta-analysis (Figure 1) analysing data from 1373 patients (see also Supplementary Table S1). Three studies analysed CD68^+^ in the stroma and intra-tumour location of OSCC. Three studies analysed CD163^+^ TAM in the stroma. 12 of the included studies were predominately performed in Asia and one in Europe. The main characteristics of eligible studies and data extraction are shown in Tables 2, 3.

**TABLE 2:** Main characteristics of included studies in meta-analysis.

Author/year [References]	Region	No. of participants	Age (mean, Range)	Location of tumour analysed	Stage
Fujii et al., 2012 (44)	Japan	108	66.4, 23–93	Tumour Stroma	I-IV
Fujita et al., 2014 (45)	China	50	68.6, 48–93	Invasive front	I-IV
Wang et al., 2014 (46)	China	298	53, 21–78	Tumour Stroma	I-IV
Matsuoka et al., 2015 (47)	Japan	60	68.9, 33–87	Tumour Stroma at invasive front	I-IV
Takahashi et al., 2017 (48)	Japan	73	69, 36–92	Tumour Stroma	I-IV
Ni et al., 2015 (49)	China	91	55, 20–78	Normal OSCC tissue, tumour nest and tumour stroma	I-IV
Fang et al., 2017 (50)	China	78	60, 24–82	Tumour stroma, tumour epithelial, advancing tumour margin	I-IV
Kikuchi et al., 2021 (51)	Japan	103	70, 30–92	Tumour Stroma, Intra-tumoural compartment	I-IV
Lin et al., 2015 (52)	Taiwan	305	N/A	Normal OSCC tissue	I-IV
Kogashiwa et al., 2017 (40)	Japan	84	68, 20–92	N/A	I-IV
Ahn et al., 2017 (53)	South Korea	68	57.7, 23–84	Normal OSCC tissue	I-IV
Lenouvel et al., 2021 (54)	Spain	55	66.8, 42–87	Tumour Stroma	I-IV

N/A, not reported.

**TABLE 3:** Data extraction from included studies related to outcomes in meta-analysis.

Author/year [References]	Biomarker	Follow-up (months)	Cut-off point	Univariate or multivariate analysis	Overall survival (HR (hazard ratio), 95% cl)
Fujii et al., 2012 (44)	CD163	N/A	Median, 1.6 HPF (high pass filter) (CD163)	Multivariate	2.64, 1.02–6.80
Fujita et al., 2014 (45)	CD163	N/A	Median	Multivariate	4.53, 0.75–27.36 (Estimated)
Wang et al., 2014 (46)	CD163	61.5 (Median)	Median	Multivariate	3.56, 1.67–7.59
Matsuoka et al., 2015 (47)	CD163	N/A	Median, 3.2 HPF (CD163)	Multivariate	2.30, 0.65–8.10
Takahashi et al., 2017 (48)	CD68, CD163	30.5 (Median)	Median, 204	Univariate (CD68)	1.11. 0.34–3.70 (CD163)
			±200 (CD68), 64 ± 55 (CD163)	Multivariate (CD163)	2.33, 1.00–5.45 (CD68)
Ni et al., 2015 (49)	CD68	N/A	≥75%	Univariate	1.39, 0.28–6.89
Fang et al., 2017 (50)	CD68	48 (Median)	Mean	Multivariate	0.73, 0.43–1.31
Kikuchi et al., 2021 (51)	CD68, PD-L1	40.8 (Median)	Median	Univariate (CD68)	0.84, 0.31–2.26 (CD68)
			≥1 and ≥20	Univariate (PD-L1)	0.50, 0.18–1.39 (PD-L1)
Lin et al., 2015 (52)	PD-L1	45.6 (Mean)	N/A	Univariate	1.21, 0.89–1.64
Kogashiwa et al., 2017 (40)	PD-L1	40.6 (Mean)	Mean	Multivariate	0.26, 0.10–0.65
Ahn et al., 2017 (53)	PD-L1	44.3 (Mean)	N/A	Univariate	0.32, 0.11–0.93
Lenouvel et al., 2021 (54)	PD-L1	56 (Median)	5% TPS (tumour proportion score)	Univariate	0.58, 0.14–2.45

N/A, not reported

### CD163^+^ TAMs are Associated With Poor Prognosis in OSCC

**FIGURE 2:**
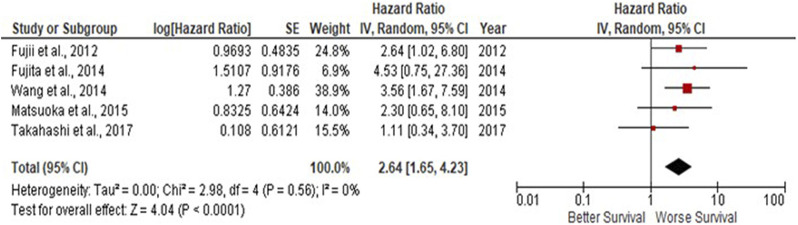
CD163^+^ TAMs are associated with poor overall survival in OSCC Forest plot reveals Hazard ratios (HRs) and 95% Cl for the association of CD163^+^ TAMs and overall survival (OS) in OSCC patients. Red square represents hazard ratio for each study, horizontal lines represent 95% confidence intervals and vertical line represents line of no effect. Black diamond represents the mean weighted overall hazard ratio among all studies (pooled estimate). An HR >1 illustrates a higher risk of death or progression associated with high CD163^+^ TAM expression. Forest plot reveals statistical significance between high CD163^+^ TAMs expression and OS in OSCC patients (*p* < 0.0001). Heterogeneity equates to 0% and results are conducted in a random-effect model.

**FIGURE 3:**
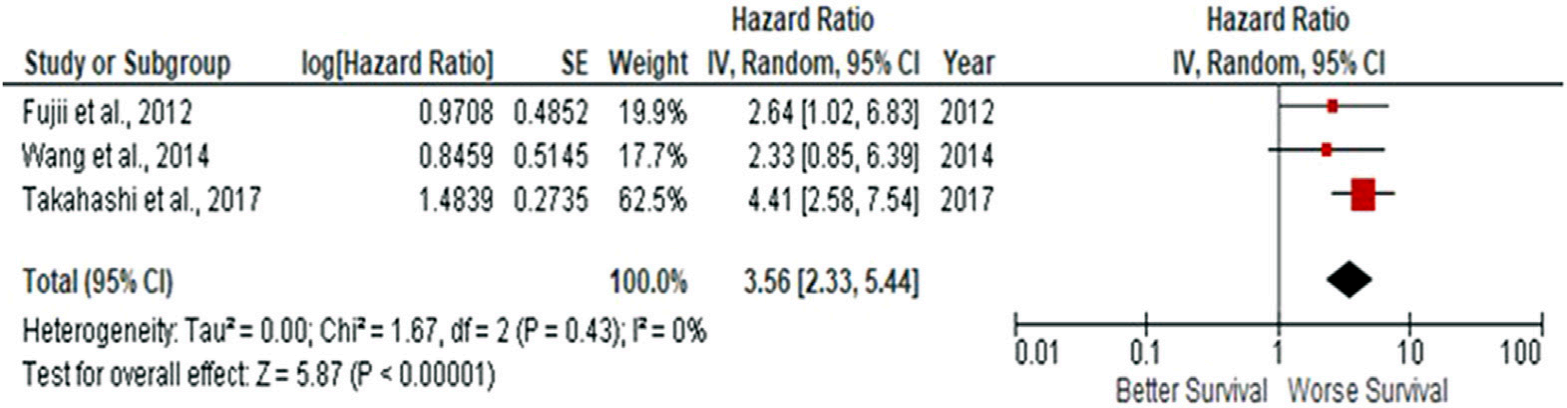
Stromal-located CD163^+^ TAMs are also associated with poor overall survival in OSCC Forest-plot reveals hazard ratios (HRs) and 95% Cl in accordance to stromal localisation of CD163^+^ TAMs in OSCC samples. Red square represents hazard ratio for each study, horizontal lines represent 95% confidence intervals and vertical line represents line of no effect. Black diamond represents the mean weighted overall hazard ratio among all studies (pooled estimate). An HR >1 illustrates a higher risk of death or progression associated with high stromal CD163^+^ TAMs. Forest plot reveals statistical significance between high CD163^+^ TAMs expression in accordance to stromal localisation in OSCC samples (*p* < 0.00001). Heterogeneity equates to 0% and results are conducted in a random-effect model.

Due to observations that M2-like CD163^+^ TAMs were associated with poor prognosis in HNSCC, breast, gastric, colorectal and hepatocellular cancers, this study investigated whether CD163^+^ TAMs could also be adopted as a prognostic indicator in OSCC. The meta-analysis was executed at a random-effect model as a result of its low rate of heterogeneity (I^2^ = 0%). Five eligible studies reported the prognostic value of CD163^+^ TAM in OSCC. The pooled analysis revealed a high expression of CD163^+^ TAM and overall survival (OS) corresponded to a worse survival in OSCC patients (HR = 2.64; 95% Cl: [1.65, 4.23]; *p* < 0.0001) (Figure 2). Furthermore, in accordance to the stromal localisation of CD163^+^ TAM, it revealed similar results with the association being significant in stromal expression in OSCC patients (HR = 3.56; 95% Cl: [2.33, 5.44]; *p* < 0.00001) (Figure 3).

### Tumour and Stromal CD68^+^ TAMs Fail to Predict Prognosis in OSCC

**FIGURE 4:**
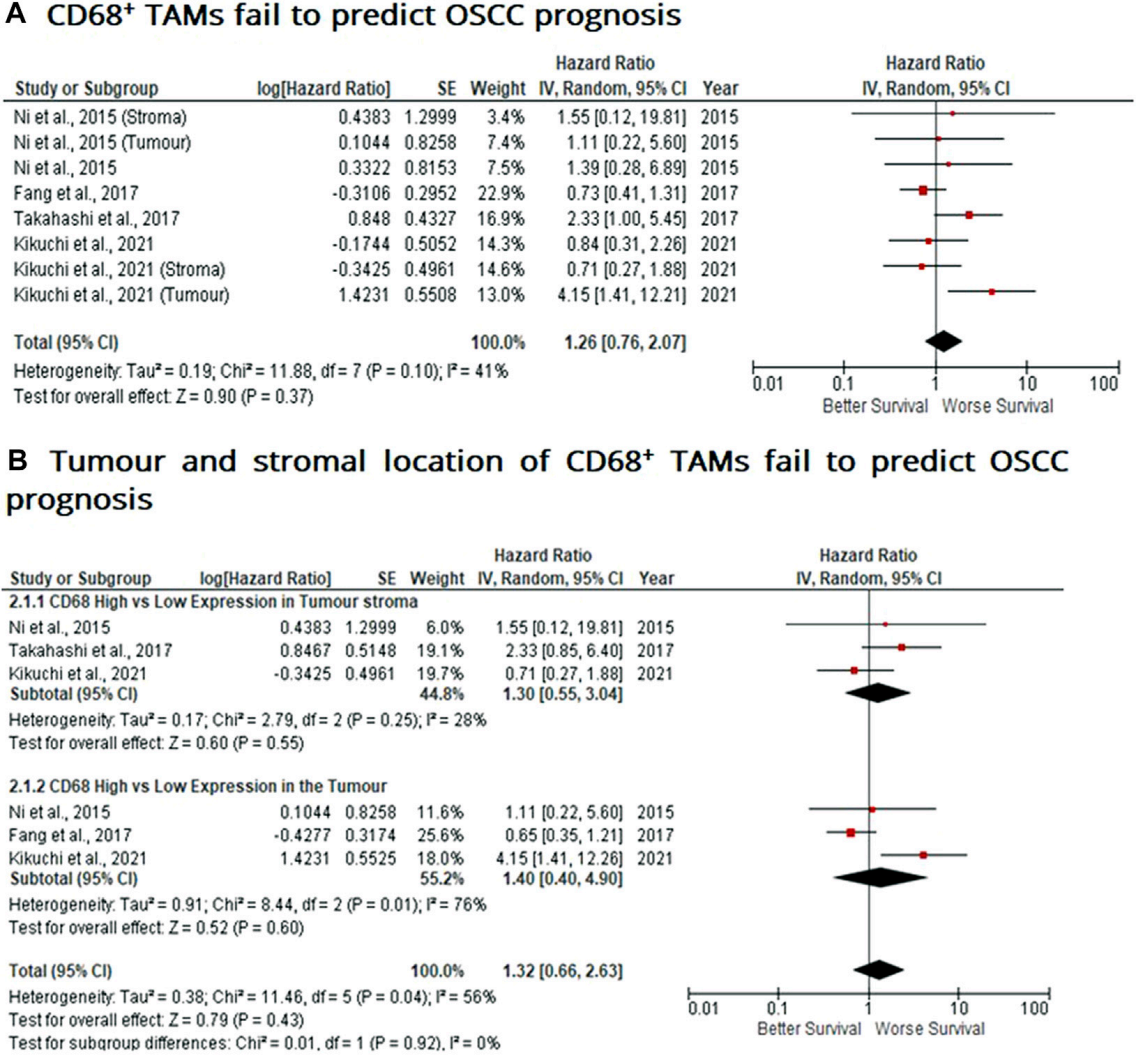
Tumour and stromal CD68^+^ TAMs fail to predict prognosis in OSCC Forest-plot reveals hazard ratios (HR) and 95% Cl for the association of CD68^+^ TAMs and OS in OSCC patients. **(A)** Studies were released evaluating the expression of CD68^+^ TAMs in several areas of the samples from the same group, whereas **(B)** is a Forest-plot presenting hazard ratios (HRs) and 95% Cl in a subgroup analysis related to survival in accordance to stromal or intra-tumour localisation of CD68^+^ TAMs in OSCC samples. Red square represents hazard ratio for each study, horizontal lines represent 95% confidence intervals and vertical line represents line of no effect. Black diamond represents the mean weighted overall hazard ratio among all studies (pooled estimate). An HR >1 illustrates a higher risk of death or progression associated with high levels of CD68^+^ TAMs. Tests for overall effect reveals statistical significance between CD68^+^ TAMs and OS in OSCC patients (*p* value) according to tumour as a whole **(A)** or specific tumour location **(B)**.

Similar to findings of M2-like CD163^+^ TAMs, the presence of CD68^+^ TAMs is associated with poor prognosis in nasopharyngeal carcinoma (NPC), gastric and hepatocellular cancers, this study investigated whether CD68^+^ TAMs could also be adopted as a prognostic indicator in OSCC. The meta-analysis was executed at a random-effect model as a result of its low rate of heterogeneity (I^2^ = 41%). Four eligible studies reported the prognostic value of CD68^+^ TAM in OSCC. It should be noted in this analysis, included studies evaluate the expression of CD68^+^ TAM in more than one area (stroma and tumour (intra-tumoural) area). The pooled analysis revealed the association between high CD68^+^ TAMs and OS showed no statistically significant difference in OSCC patients (HR = 1.26; 95% Cl: [0.76, 2.07]; *p* = 0.37) (Figure 4A). In addition, CD68^+^ TAM expression was evaluated in different sample locations (stroma vs. tumour). The subgroup analysis revealed no association between stromal (HR = 1.30; 95% Cl: [0.55, 3.04]; *p* = 0.55) or tumour (intra-tumoural) (HR = 1.40; 95% Cl: [0.40, 4.90]; *p* = 0.60) expression of CD68^+^ TAMs and OS in OSCC patients (Figure 4B).

### PD-L1 Expression May be Associated With a Positive Prognosis in OSCC

**FIGURE 5:**
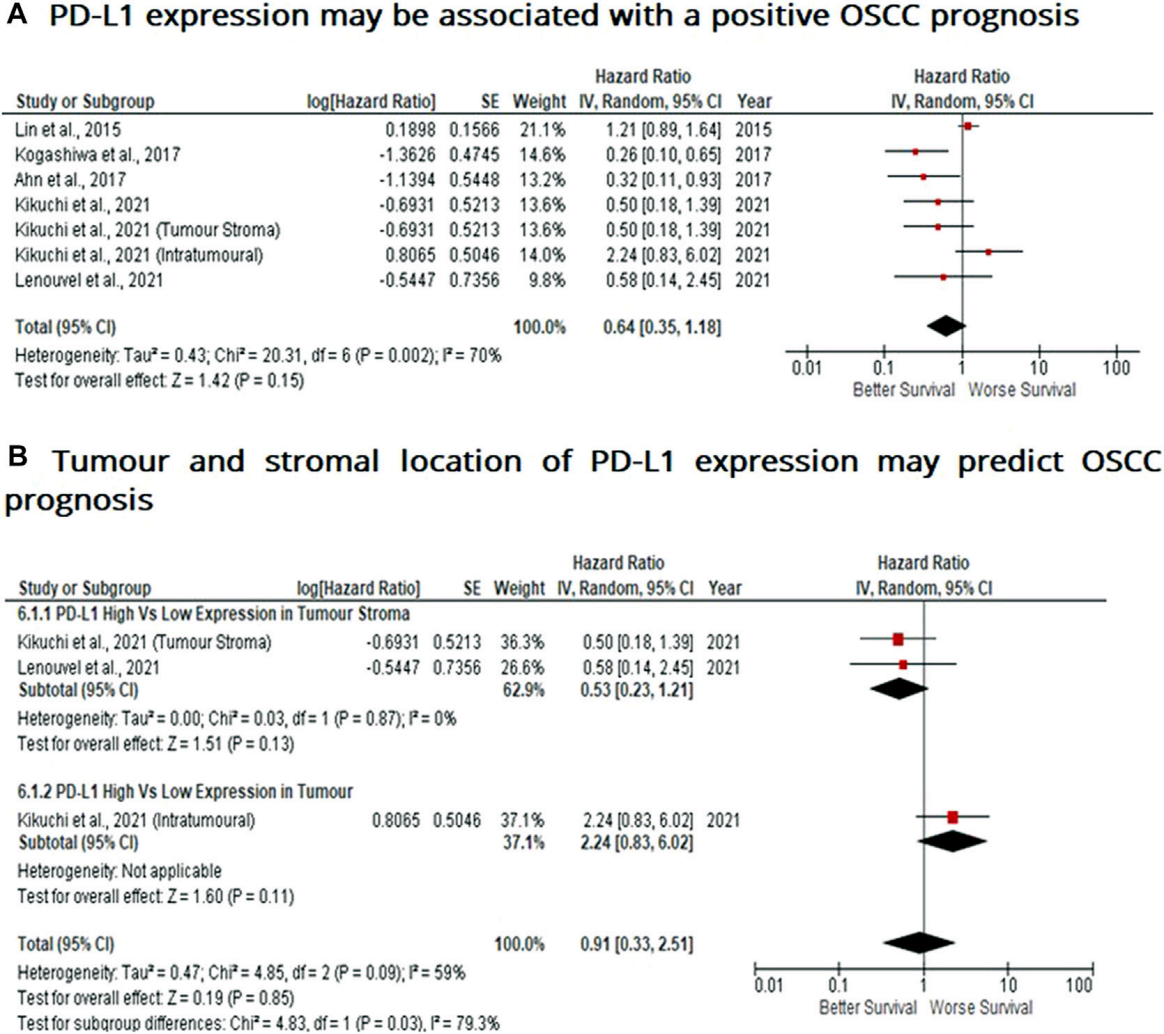
PD-L1 expression may be associated with a positive prognosis in OSCC Forest-plot reveals hazard ratios (HRs) and 95% Cl for the association of PD-L1 expression and OS in OSCC patients. **(A)** Studies evaluating the expression of PD-L1 in several areas of tumour samples from the same group, whereas **(B)** Forest-plot reveals hazard ratios (HRs) and 95% Cl in a subgroup analysis related to survival in accordance to stromal or intra-tumour localisation of PD-L1 in OSCC samples. Red square represents hazard ratio for each study, horizontal lines represent 95% confidence intervals and vertical line represents line of no effect. Black diamond represents the mean weighted overall hazard ratio among all studies (pooled estimate). An HR <1 illustrates a better overall survival associated with PD-L1 expression. Tests for overall effect reveals statistical significance between PD-L1 expression and OS in OSCC patients (*p* value) according to tumour as a whole **(A)** or specific tumour location **(B)**. Percentage heterogeneity (I^2^) is indicated and as such, results are conducted in a random-effect model.

When considering the immune-suppressive nature of PD-L1, it was not surprising to note that this marker was associated with a poor prognosis in breast, bladder and non-small cell lung cancer as well as in malignant pleural mesothelioma and renal cell carcinoma, whereas the opposite prognosis was observed in breast cancer and HKSCC. The present study wished to investigate whether PD-L1 acted as either a positive or negative prognostic indicator. Five eligible studies reported the prognostic value of PD-L1 expression in OSCC. A high rate of heterogeneity was found (I^2^ = 70%), therefore a random effect model was performed. In this analysis, one study evaluated PD-L1 expression twice (two areas of the sample from the same cohort) was included. Pooled analysis revealed the association of high PD-L1 expression and OS showed no statistically significant difference in OSCC patients (HR = 0.64; 95% Cl: [0.35, 1.18]; *p* = 0.15) (Figure 5A). In addition, PD-L1 expression was evaluated in different sample locations (stroma vs. tumour). The subgroup analysis revealed no association between stromal (HR = 0.53; 95% Cl: [0.23, 1.21]; *p* = 0.13) or tumour (intra-tumour) (HR = 2.24; 95% Cl: [0.83, 6.02]; *p* = 0.11) expression of PD-L1 and OS in OSCC patients (Figure 5B).

## Discussion

This meta-analysis has reviewed the current literature on the prognostic potential of tissue biopsy tumour-associated macrophages; CD163^+^ TAMs and CD68^+^ TAMs as well as PD-L1 expression in OSCC. This meta-analysis demonstrated a significant association between high CD163^+^ TAMs with poor survival/prognosis in OSCC patients. Additional results revealed an insignificant association of CD68^+^ TAMs with OS, whereas PD-L1 expression approaches significance, indicating a potential positive prognosis associated with OSCC patients. Therefore, it reveals CD163^+^ TAMs to exhibit the best prognostic potential of macrophage subsets in both intra-tumour and stromal OSCC biopsies.

The present pooled analysis revealed a high density of CD163^+^ TAMs was associated with worse overall survival in OSCC (HR = 2.64; 95% Cl: [1.65, 4.23]; *p* < 0.0001). These findings are consistent in several other studies, reporting the correlation of CD163^+^ macrophages and worse survival in breast, gastric, colorectal and hepatocellular cancers (24, 55–57). Also, pooled analysis found CD163^+^ located within the tumour stroma to be associated with poor survival in OSCC patients (HR = 3.56; 95% Cl: [2.33, 5.44]; *p* < 0.00001). These findings were consistent with high CD163^+^ TAM densities in the tumour stroma and associated with poor survival in SCCHN (squamous cell carcinoma of the head and neck) (26). Similarly, high levels of tumour stroma CD163^+^ TAMs were associated with lymph node metastasis in OSCC (58). These high levels of OSCC CD163^+^ TAMs could be explained by the ability of TAMs to directly stimulate EGF (epidermal-growth factor) as well as anti-inflammatory cytokines (such as IL-6, IL-10), pro-inflammatory cytokines such as TNF-α, and chemokines such as CXCL12, CCL16, CCL18. Collectively, these factors induce tumour cell growth and survival factors which enhance tumour cell proliferation, migration and metastasis (59, 60).

Interestingly, the expression of CD163 is not only restricted to TAMs but may also be associated with cell fusion where the fusion of cancer cells and TAMs can increase metastatic potential with migratory leukocytes in cancer patients and plays a role in cancer progression. This can lead to a more aggressive and metastatic phenotype causing an unfavourable prognosis as demonstrated in OSCC (61, 62). Therefore, it is imperative to discriminate CD163^+^ malignant cells and macrophages when examining the influence of CD163^+^ on prognosis. Nevertheless, many studies reveal consistent findings with the present results demonstrating CD163 may serve as a significant prognostic biomarker in OSCC. Interestingly, the results presented about CD163^+^ TAMs may provide clinical implications. More specifically, they may serve as therapeutic targets for anticancer therapeutic regimens which may include the repolarisation of TAMs from M2-like TAMs to an M1-like phenotype, to restrain tumour progression (63). Therefore, a greater understanding of TAM function and OSCC progression is critical for future research in TAM-targeted therapies.

Compared to CD163^+^ TAMs, pooled results demonstrated high CD68^+^ TAMs were not statistically significant in revealing poor overall survival in OSCC patients (HR = 1.26; 95% Cl: [0.76, 2.07]; *p* = 0.37). However, other meta-analysis, has revealed high CD68^+^ TAM densities were associated with worse overall survival and disease-free survival in nasopharyngeal carcinoma (NPC) patients (64). Similar studies revealed CD68^+^ TAMs were associated with poor survival in gastric cancer and hepatocellular carcinoma, respectively (65, 66). These findings may be indicative of CD68^+^ TAMs possessing immunosuppressive and pro-tumour responses, favouring cancer progression. Interestingly, CD68^+^ TAMs have been shown to suppress cytotoxic activity of CD8^+^ T-cells and increase tumour growth (67). On the other hand, in the case of oesophageal squamous cell carcinoma, CD68^+^ TAMs were correlated with a favourable prognosis (68), suggestive that CD68^+^ TAMs may also function as M1 macrophages, revealing pro-inflammatory and anti-tumour effects. Whilst these molecular mechanisms are not fully understood, studies reveal TAMs may exert tumoricidal activity *in vitro;* more specifically, to polarise into M1 TAMs orchestrated by the production of IFN-γ which also activates cytotoxic CD8^+^ T and NK cell responses to initiate tumour cell killing (12, 19).

Similar to findings in this study, CD68^+^ TAMs did not significantly correlate with overall survival and recurrence-free survival (RFS) in multivariate analysis in basal-like breast cancer (BLBC) and triple-negative cancer of the breast (24, 69). In addition, no prognostic utility was found between CD68^+^ TAMs and OS in SCCHN patients (26). These findings reveal CD68^+^ TAMs may serve as a poor prognostic biomarker as demonstrated in this study focussed on OSCC, but more importantly, may indicate CD68 as a pan-macrophage marker expressed by both M1-like and M2-like TAMs, capable of exhibiting opposing effects on the tumour microenvironment (70). Therefore, this study’s findings and the mounting conflicting evidence between different cancers, indicates that CD68^+^ TAMs may be a poor prognostic biomarker in OSCC or at least requires further investigation across a variety of cancers/tumours as well as their TMEs.

Contrary to expectation, pooled results also revealed high PD-L1 expression had a non-significant positive impact on overall survival in OSCC patients (HR = 0.64; 95% Cl: [0.35, 1.18]; *p* = 0.15). A high level of heterogeneity (I^2^ = 70%) among the included studies were revealed, demonstrating conflicting results with each other. In contrast to these data however, numerous studies reveal PD-L1 expression was associated with poor prognosis and overall survival (OS) in solid cancers, such as head and neck squamous cell carcinoma (HNSCC) (71), breast cancer (72), non-small cell lung cancer (NSCLC) (73) and bladder cancer (74). This association with poor prognosis may be suggested by PD-L1/PD-1 binding to suppress CD8^+^ cytotoxic T-lymphocyte activation, leading to the evasion of the host immune anti-tumour response, thereby decreasing the survival rate in many cancers (37, 75). Additionally, whilst PD-L1 expression may protect macrophages from cell death, OSCC tumour cells induce TAM PD-L1 expression *via* IL-10 and induce T-cell apoptosis, further reinforcing an unfavourable prognosis (76).

Contradictory to these studies, yet consistent with findings in this investigation, PD-L1 expression in primary tumour cells was associated with prolonged DFS (Disease-free survival) in HNcSCC (head and neck cutaneous squamous cell carcinoma) (77). Similarly, high PD-L1 expression correlated better OS and DFS in breast cancer patients (78). This observation in prolonging survival in patients with PD-L1 expression may be due to the induction of an anti-tumour immune response. More specifically, IFN-γ, released by tumour-infiltrating lymphocytes as an adaptive immune-resistance mechanism to inhibit local effector T-cell function, can upregulate PD-L1 expression in tumour cells (36). Interestingly, IFN-γ also induces protein kinase D isoform 2 (PKD2), an important negative regulator of PD-L1 expression in OSCC. Thus PDK2 inhibits PD-L1 expression and promotes anti-tumour effects (blocking PD-1/PD-L1 dependent tumour antigen-specific CD8^+^ T cell apoptosis) (79). In addition, a high expression of PD-L1 was not statistically associated with OS in oesophageal squamous cell carcinoma (80), and more recently, pooled analysis of high PD-L1 expression did not have a statistically significant association with OS, DFS, DSS (Disease-specific survival) in OSCC patients (81). The findings of this investigation (Figure 5) are consistent with these studies focussed on OSCC and oesophageal SCC. In addition, further subgroup analysis suggested that stromal expression of PD-L1 may be associated with improved survival, whereas intra-tumour PD-L1 expression may be associated with poor prognosis and overall survival. This may be indicative of PD-L1^+^ cell location is predictive of survival and may reflect stage of cancer, or, when contrasted with the poor survival observed for CD163^+^ TAMs, is suggestive that stromal PD-L1 and CD163 are expressed on different TAM subsets or that PD-L1 may not be expressed on TAMs at all. Thus, this current investigation goes some way to indicating PD-L1 as a prognostic marker of survival, or indeed stage of cancer progression in OSCC which may reach statistical significance with the inclusion of more clinical studies. Further investigation may also potentially validate PD-1/PD-L1 interaction as a future therapeutic target for OSCC.

## Conclusion

In conclusion, this meta-analysis confirmed the prognostic role of CD163^+^ TAMs, where a high cell number was associated with poor overall survival in OSCC. This indicates CD163^+^ TAMs may be a useful novel prognostic biomarker for OSCC and may suggest TAMs as a potential therapeutic target. Both CD68^+^ TAMs and PD-L1 revealed an insignificant correlation with overall survival in OSCC patients and limits the prognostic value of both biomarkers in OSCC, however the fact that the OS approached significance for PD-L1 is potentially indicative of PD-L1 being revealed as a positive prognostic indicator in the future.

## Summary Table

### What is Known About Subject

• Presence of TAMs are associated with poor prognosis of tumours including OSCC.

### What This Paper Adds

• In contrast to other cancers, CD68^+^ TAMs fail to indicate OSCC prognosis.
• CD163^+^ TAMs and expression of PD-L1 could serve as both prognostic indicators of survival and stage of tumour progression: counter-intuitive, as these markers are normally associated with M2 subset, which is described as pro-tumoral.
• TAM subset analysis and location (tumour or stroma) is indicative of OSCC stage and prognosis.

## Summary Sentence

• TAMs, and their location, are indeed, indicative of OSCC survival; where both tumour and stromal located CD163^+^ TAMs are indicative of poor prognosis whereas stromal PD-L1 expression may be indicative of a better prognosis when compared to tumour expressed PD-L1.

## Data Availability

The original contributions presented in the study are included in the article/Supplementary Material, further inquiries can be directed to the corresponding author.
