# Supplementary material for: Prognostic Role of CD68+ and CD163+ Tumour-Associated Macrophages and PD-L1 Expression in Oral Squamous Cell Carcinoma: A Meta-Analysis
Source: Br J Biomed Sci. 2023 Jun 16;80:11065. doi: 10.3389/bjbs.2023.11065 (PMC10310926; doi:10.3389/bjbs.2023.11065)
Supplement: Supplementary file 1 [file DataSheet1.docx]

***Supplementary Material***

**Supplementary table 1.** Data extraction for 12 selected publications.

| **Study** | **Main Topic** | **Read outs** |  | **Outcome** | **Characteristics** |  |  |  |  |  |  |  |  |  |  |  |  |  |  | **Key Words** | **Search Engine** |
| --- | --- | --- | --- | --- | --- | --- | --- | --- | --- | --- | --- | --- | --- | --- | --- | --- | --- | --- | --- | --- | --- |
|  |  | **Description** | **Numerical Data** |  | **Year** | **Region** | **Participants** | **Male** | **Female** | **Age** | **Type of Cancer** | **Stage** | **Location of tumour analysed** | **Overall Survival (OS) analysis** | **Disease-free survival (DFS) analysis** | **Progression-free survival (PFS) analysis** | **Disease-specific survival (DSS) analysis** | **Detection Method** | **Marker** |  |  |
| **Fang et al., 2017** | Prognostic significance of tumour infiltrating immune cells in oral squamous cell carcinoma | CD57 and CD8 showed longer overall survival  CD57 cells showed no metastasis in lymph node  CD57 and CD8 shows high levels of prediction  Kaplan-Meier curves for overall survival indicated that higher CD8 and CD57 expression were associated with better patient survival | Mean number of immune cells CD8, CD4, CD68, CD57 were 28.99, 62.06, 8.97, 21.25 and 15.75 respectively in 78 OSCC samples  average overall survival of patients were 21.38 months in low and 43.68 months in high CD8 expression group  Average overall survival of patients were 22.14 months in low and 46.2 months in high CD57 expression group  AUCs in ROC curve of CD8 and CD57 were 0.784 and 0.868 respectively, significantly higher than TNM staging (0.599)  D8 mean cells/hpf = 28.99 (12.67)  CD4 mean cells/hpf = 62.06(21.33)  T-bet mean cells/hpf = 8.97(3.99)  CD68 mean cells/hpf = 21.25 (6.01)  CD57 mean cells/hpf = 15.75 (9.41) | Tur stroma CD57 and CD8 expression associated with lymph node status is able to predict survival of OSCC patients | 2017 | China | 78 | 57 | 21 | 24-82 | Oral Squamous Cell Carcinoma | Tumour Stage: T1-T4  Clinical Stage: I - IV  N stage:  N0 - N3 | Tumour stroma  Tumour cell nests  Tumour epithelial  Tumour margin | Univariate analysis:  CD8:  (95% Cl) = 1.998 7.256  CD4 (95% Cl) = 0.382 1.232  T-bet (95% Cl) = 0.489 1.551  CD68:  HR = 0.733  (95% Cl) = 0.411 1.308  CD57 (95% Cl) = 3.646 16.338    Multivariate analysis:  CD8:  (95% Cl = 1.078 - 4.384  CD4 (95% Cl) = 0.545-1.977  T-bet (95% Cl) = 0.580-1.959  CD68 (95% Cl) = 0.825-2.848  CD57 (95% Cl) = 2.678-15.623 | ? | ? | ? | IHC | CD8, CD4, CD68, CD57 | ? | Pub-Med |
| **Fujii *et al.,* 2012** | Cancer-associated fibroblasts and CD163- positive macrophages in oral squamous cell carcinoma: their clinicopathological and prognostic significance | Clinicopathological findings shows that grade 2 CAF group was significantly increased compared to Grade 0/1 group  Grade 2 CAFs and high CD163-positive macrophage levels significantly correlated with a poor outcome in patients with OSCC | mean (median) number of CD68+ and CD163+ macrophages at the invasive areas of the OSCC specimens was 2.72 ± 3.53 (1.67) and 2.29 ± 3.38 (1.33) respectively | Cancer associated fibroblasts and CD163- positive macrophages may be potential prognostic predictors of OSCC and associated with cancer invasion. | 2012 | Japan | 108 = OSCC  24 = oral dysplasia  5 normal oral epithelial specimens | OSCC = 67  Oral dysplasia = 6  Normal oral epithelia = ? | OSCC = 41  Oral dysplasia = 18  Normal oral epithelia = ? | 23-93 | Oral Squamous Cell Carcinoma | Clinical Stage: I - IV  Tumour Stage: T1-T4  N stage: N0-N3 | Tumour stroma | - - CD163+ (Low vs High):   Hazard ratio = 2.636  95% Cl = 1.021-6.083  *P =* 0.045 | ? | ? | ? | IHC | CD163 and CD68 |  | Pub-Med |
| **Ni *et al.,* 2015** | Microlocalization of CD68+ tumour-associated macrophages in tumour stroma correlated with poor clinical outcomes in oral squamous cell carcinoma patients | The number of CD68+ TAMs was remarkably increased from adjacent none-neoplasia tissues (NT) to tumour nest (TN), but tumour stroma (TS) was infiltrated with highest frequency of CD68+ TAM.  More CD68+ TAMs in TS but not NT or TN, were associated with high tumour grade, lymph node metastasis and shorter disease free survival  High infiltration group of TAM in TS compartments correlated with poor overall survival and disease free survival | Mean number of CD68+ cells in NT, TN, and TS was 6.4 ± 5.5 cells, 34.3±13.9 and 56.7±24.9 cells respectively. | CD68+ TAM in TS was linked to higher TNM stage, LNM, and short OS or DFS  Reduced number of monocytes and lymphocytes correlated to high infiltrated CD68+ TAMs in TS  Our results suggest that microlocalisation of CD68+ TAMs in tumour stroma predicted poor clinical outcome for OSCC patients | 2015 | China | 91 | 38 | 53 | 0-55 | Oral Squamous Cell Carcinoma | TNM stage: I - IV | Tumour nest  Tumour stroma | CD68 in Normal tissue:  Hazard ratio = 0.717  95% Cl = 0.145-3.541  *P* = 0.846  CD68 in tumour stroma:  Hazard ratio = 1.947  95% Cl = 1.512 - 10.379  *P =* 0.033  CD68 in tumour nest:  Hazard ratio = 0.904  95% Cl = 0.180 - 4.552  *P =* 0.802 | ? | ? | ? | IHC | CD68 | Microlocalisation  CD68  Tumour associated macrophages  Prognosis  Oral Squamous Cell Carcinoma | Pub-Med |
| **Fujita et al., 2014** | Prognostic Significance of Interleukin-8 and CD163-positive cell infiltration in tumour tissues in patients with Oral Squamous Cell Carcinoma | Disease free survival (DFS) was significantly lower in the stage I/II OSCC patients with low serum IL-8 levels compared to those with higher levels  Patients with low CD163 (invasive front) showed longer OS and DFS compared to patients with high CD163 (IF).  Tumour expression of IL-8 and density of CD163+ cells in the tumour invasive front were correlated with the serum IL-8 levels and associated with poor clinical outcome in all patients  Combination of N status with IL-8(T) and CD163(IF) may be a new criterion for discriminating between OSCC patients at high and low risk for tumour relapse | ? | IL-8 may be involved in poor clinical outcomes via generation of CD163+ M2 macrophages and these factors may have prognostic value in patients with resectable OSCSS. | 2014 | Japan | 50 | 32 | 18 | 48-93 | Oral Squamous Cell Carcinoma | Tumour stage = I-IV  N Stage =N0- N3 | Tumour stroma | ? | CD163 (High vs Low):  HR = 2.625  95% Cl = 1.312-5.253  P = 0.006 | ? | ? | IHC | CD163 |  | Web of Science |
| **Lin et al., 2015** | High PD-L1 Expression Correlates with Metastasis and poor prognosis in oral squamous cell carcinoma | Advanced tumours were also more prevalent than early tumours in male patients (male vs. female, 59.8% vs. 33.3% for stage IV, P<0.0001; 69.1% vs. 46.4% for stage III+IV, P = 0.0006; 53.8% vs. 27.5% for T3+T4, P = 0.0001)  Overall survival data showed death was a more common outcome in male than in female OSCC patients (62.3% Male vs 24.6% Female P<0.0001)  High PD-L1 cytoplasm intensity was more likely in tumours from female than from male patients (staining intensity: 0–1, 60.6% for male vs. 42.0% for female; staining intensity: 2–3, 58.0% for female vs. 39.4% for male, P = 0.0062)  High expression levels of PD-L1 were also more likely to occur in tumours from female than from male patients  High PD-L1 expression (staining intensity: 2-3) was significantly associated with distant metastasis  PD-L1 expression was not associated with the death rate of patients in terms of overall survival.  Male gender, smoking and advanced stage (clinicopathological characteristics) with poor clinical outcome in univariate analysis  High PD-L1 expression was significantly associated with poor prognosis in male patients and smoking patients in multivariate analysis. | PD-L1 Expression:  Low:  Male: 143 (83.1)  Female: 29 (16.9)  I+II tumour stage: 61 (55.5)  III+IV tumour stage: 111 (56.9)  Overall Survival: Alive (87 (61.7))/Dead (85(51.8))  High:  Male: 93 (69.9)  Female: 40 (30.1)  I+II tumour stage: 49 (44.5)  III+IV tumour stage: 84(43.1)  Overall Survival: Alive 54(38.3) /Dead (79)(48.2)) | Results suggested that patients with high PD-L1 expression had poor clinical outcome and might require PD-L1-targeted immunotherapy to improve their prognosis | 2015 | Taiwan | 305 | ? | ? | ? | Oral Squamous Cell Carcinoma | Stage: I-IV  T value: T1-T4  N value: N0 - N2  Distant Metastasis: M0-M1 | ? | PD-L1 Expression  High/Low:  HR = 1.209  95% Cl = 0.890-1.643  *P = 0.2254* | ? | ? | ? | IHC | PD-L1 | ? | PubMed |
| **Kikuchi et al., 2021** | Clinical significance of tumour-associated immune cells in patients with oral squamous cell carcinoma | PD-L1 was expressed in 37 patients (36%)  The median number of total PD‐L1, PD‐1, CD3, CD4, CD8, and CD68‐positive TAICs was 10, 20, 80, 10, 70, and 50, respectively.  The median number of intratumoural PD‐L1, PD‐1, CD3, CD4, CD8, and CD68‐positive TAICs was 0, 5, 12, 2, 10, and 10, respectively, whereas that of stromal miPD‐L1, PD‐1, CD3, CD4, CD8, CD68‐positive TAICs was 10, 15, 60, 10, 50, and 30, respectively.  Stromal miPD‐L1, PD‐1, CD3, CD4, CD8, and CD68‐positive TAICs were significantly more predominant than intra‐tumoral | PD-L1 expression in immunohistochemistry:  85% (88/103) (cut off >1)  15% (15/103) (cut off >20)  Median number of total in IHC:  PD-L1 = 10  PD-1 = 20  CD3 = 80  CD4 = 10  CD8 = 70  CD68 = 60  Median number of intratumoural IHC:  PD-L1 = 0  PD-1 = 5  CD3 = 12  CD4 = 2  CD8 = 10  CD68 = 10  Median number if stroma in IHC:  PD-L1 = 10  PD-1 = 15  CD3 = 60  CD4 = 10  CD8 = 50  CD68 = 30 | PD-1 and CD68 in the intratumoural area revealed as positive and negative prognostic markers of OS for OSSC patients, respectively. | 2020 | Japan | 103 | 60 | 43 | 30-92 | Oral Squamous Cell Carcinoma | - - Tumour Classification: T1-T4   - Nodal classification: N0-N3   - Stage: I-IV | Intratumoural area  Tumour stroma | Total immune marker expression:  PD-L1(High vs Low):  Univariate:  HR: 0.50  95% Cl: 0.18-1.41  P = 0.19  PD-1(High vs Low):  Univariate  HR: 0.28  95% Cl: 0.08-0.97  P = 0.04  Multivariate:  HR = 0.20  95% Cl = 0.05-0.78  P = 0.02  CD3(High vs Low):  Univariate  HR: 0.69  95% Cl: 0.26-1.88  P = 0.47  CD4(High vs Low):  Univariate:  HR: 0.50  95% Cl: 0.18-1.41  P = 0.19  CD8(High vs Low):  Univariate:  HR: 0.72  95% Cl: 0.27-1.94  P = 0.51  CD68(High vs Low):  Univariate:  HR: 0.84  95% Cl: 0.31-2.27  P = 0.73    Intratumoural immune marker expression:  PD-L1(High vs Low):  Univariate:  HR: 2.24  95% Cl: 0.83-6.06  P = 0.11  PD-1(High vs Low):  Univariate  HR: 0.53  95% Cl: 0.19-1.51  P = 0.24  CD3(High vs Low):  Univariate  HR: 0.65  95% Cl: 0.25-1.71  P = 0.38  CD4(High vs Low):  Univariate:  HR: 0.39  95% Cl: 0.13-1.18  P = 0.09  Multivariate:  HR: 0.34  95% Cl: 0.09-1.35  P = 0.13  CD8(High vs Low):  Univariate:  HR: 0.49  95% Cl: 0.16-1.51  P = 0.21  CD68(High vs Low):  Univariate:  HR: 2.43  95% Cl: 0.90-6.58  P = 0.08  Multivariate:  HR: 4.15  95% Cl: 1.36-12.7  P = 0.01    Stromal immune marker expression:  PD-L1(High vs Low):  Univariate:  HR: 0.50  95% Cl: 0.18-1.41  P = 0.19  PD-1(High vs Low):  Univariate  HR: 0.65  95% Cl: 0.24-1.76  P = 0.39  CD3(High vs Low):  Univariate  HR: 1.78  95% Cl: 0.67-4.72  P = 0.25  CD4(High vs Low):  Univariate:  HR: 0.68  95% Cl: 0.24-1.94  P = 0.48  CD8(High vs Low):  Univariate:  HR: 2.29  95% Cl: 0.84-6.26  P = 0.11  CD68(High vs Low):  Univariate:  HR: 0.71  95% Cl: 0.26-1.9  P = 0.49 | ? | ? | ? | IHC | PD-L1  PD-1  CD3  CD4  CD8  CD68 | ? | Scopus |
| **Kogashiwaet al., 2017** | PD-L1 expression confers better prognosis in locally advanced oral squamous cell carcinoma (LAOSCC) | PD-L1 and PD-L2 were expressed in 52.4% and 23.8% in LAOSCC cases  PD-L1 positivity was significantly associated with disease free (p= 0.024) and overall survival (p= 0.008) of LAOSCC patients | PD-L1 Expression:  Low:  Negative = 29  Positive = 13  High:  Negative = 11  Positive = 31  PD-L2 Expression:  Low:  Negative = 33  Positive = 9  High:  Negative = 31  Positive = 11 | PD-L1 expression was associated with CD8+ tumour-infiltrating lymphocytes and a better outcome in patients with LAOSCC | 2017 | Japan | 84 | 57 | 27 | 20-92 | Locally advanced oral squamous cell carcinoma | Tumour stage: I-IV  Nodal stage: N0-N2 | Tongue  Floor of the mouth  Buccal  Gingiva  Hard Palate  Cheek lining | Univariate analysis:  PD-L1+:  HR = 0.257  95% Cl = 0.102-0.649  P = 0.006  PD-L2+:  HR = 0.442  95% Cl = 0.132-1.486  P = 0.187  CD8 (High):  HR = 0.499  95% Cl = 0.214-1.16  P = 0.109  Multivariate anal  PD-L1+:  HR = 0.256  95% Cl = 0.101-0.646  P = 0.008 | ? | Univariate analysis:  PD-L1+:  HR = 0.576  95% Cl = 0.274-0.956  P = 0.0372  PD-L2+:  HR = 1.01  95% Cl = 0.431-2.37  P = 0.978  CD8 (High):  HR = 0.717  95% Cl = 0.342-1.50  P = 0.378  Multi-variate analysis:  HR = 0.541  95% Cl = 0.278-0.894  P = 0.0315 | ? | IHC | PD-L1  PD-L2  CD8 | ? | PubMed |
| **Wang et al., 2014** | Expression of CD163, interleukin-10 and interferon-gamma in oral squamous cell carcinoma: mutual relationships and prognostic implications | Oral squamous cell carcinoma with lymph node metastasis had significantly higher CD163 staining than did tissues without lymph node metastasis, although the histopathological grading and clinical stage did not differ between groups  There was a correlation between IFN‐*γ* and IL‐10 in patients with non‐lymph node metastasis (*P*= 0.000, *γ*= 0.873), and there was no correlation between IFN‐*γ* and IL‐10 in patients with lymph node metastasis (*P*= 0.110, *γ*= 0.426).  There was a correlation between IL‐10 and CD163 (*P*= 0.015, *γ*= 0.7333).  Using the median value as a cut‐off, patients were classified into four groups: I, low IFN‐*γ*, high IL‐10, and low CD163 (*n* = 25); II, low IFN‐*γ*, low IL‐10, and low CD163 (*n* = 20); III, high IFN‐*γ*, low IL‐10, and low CD163 (*n* = 6); and IV, high IFN‐*γ*, high IL‐10, and low CD163 (*n* = 45). Overall survival significantly differed between these groups | Normal Oral Mucosa:  IFNY = 299 ioD (integral optical density)  IL-10 = 150 ioD  CD163 = 10 ioD  Simple Hyperplasia:  IFNY = 340 ioD  IL-10 = 290 ioD  CD163 = 30 ioD  Dysplasia:  IFNY = 390 ioD  IL-10 = 400 ioD  CD163 = 50 ioD  Carcinoma in situ:  IFNY = 390 ioD  IL-10 = 410 ioD  CD163 = 100 ioD  Non-metastatic oral squamous cell carcinoma:  IFNY = 390 ioD  IL-10 = 410 ioD  CD163 = 200 ioD  Metastatic OSCC:  IFNY = 310 ioD  IL-10 = 420 ioD  CD163 = 400 ioD  Lymph node metastasis:  CD163:  Yes: 54/100  No: 42/44  P - 0.037  Death:  CD163:  Yes: 15/66  No: 81/78  P= 0.000 | We found that high levels of CD163, or a combination of low IFN‐*γ* levels, high IL‐10 levels, and low CD163 levels, were associated with poorer overall survival (OS).  CD163+ cells provide better predictive power for OS in comparison with traditional markers, such as clinical stage and lymph node metastasis.  Therefore, CD163+ cells may be effective prognostic predictors of OSCC. IL‐10 may also indicate poor outcomes when IFN‐*γ* secretion is low and the cells are CD163 | 2014 | China | 240 | ? | ? | 21-78 | Oral squamous Cell Carcinoma | Lymph Node metastasis  Clinical stage: I-IV | Oral Mucosa  Tumour stroma  Cytoplasm of tumour cells  dysplasia | CD163 (Low vs High):  UNVARIATE ANALYSIS:  HR = 4.41  95% Cl = 2.578-7.547  P= 0.00  MULTIVARIATE ANALYSIS:  HR = 3.561 95% Cl = 1.733-7.320  P= 0.001 | ? | ? | ? | IHC | IFNY  IL-10  CD163 | ? | SCOPUS |
| **Ahn et al., 2017** | Clinicopathologic implications of the miR-197/PD-L1 axis in oral squamous cell carcinoma | PD-L1 levels correlated inversely with miR-197 but correlated positively with TILs. The aggressive features of OSCC, including high stage, angiolymphatic invasion, perineural invasion, and death, were associated with TIL depletion.  High T stage (T4) tumours also had low PD-L1 but had high miR-197 expression.  In a univariate survival analysis of the full cohort, high miR-197 was associated with poor overall survival, whereas high PD-L1 expression (2+) associated with good overall survival.  In a multivariate analysis stratified based on miR-197 (median), high PD-L1 expression (2+) was an independent favourable prognostic factor for overall survival (*P* = 0.040) in the miR-197high subgroup but not the miR-197low subgroup. | Alive = 45 patients  Death = 23 patients  Mean±SD CD3+:  Alive = 47.3±40.7  Death = 33.4±27.1  Mean±SD CD4+:  Alive = 37.4±29.2  Death = 23.1±17.9  Mean±SD CD8+:  Alive = 30.4±21.7  Death = 21.9±22.6  Mean±SD PD-1:  Alive = 6.3±5.4  Death = 7.8±9.2  Mean±SD FoxP3:  Alive = 10.1±9.4  Death = 8.0±9.6  Mean±SD CD20+:  Alive = 24.6±25.7  Death = 16.3±16.70  Mean±SD miR-197:  Alive = 5.1±8.2  Death = 19.1±41.6 | This study showed inverse correlation and prognostic effects between miR-197 and PD-L1 expression in OSCC. In addition, we observed that PD-L1 expression on IHC is associated with increased TILs and favourable prognosis in miR-197high subgroup. | 2017 | South Korea | 68 | 45 | 23 | 23-84 | Oral squamous cell carcinoma | Tumour stage: T1-T4  Nodal Stage: N0-N2c  Tumour-nodal metastasis stage: I-IV | Normal OSCC tissue | Univariate analysis:  PD-1 Cell number (high):  HR = 1.01  95% Cl = 0.95-1.06  *P =* 0.826  miR-197 expression (high):  HR = 1.01  95% Cl = 1.00-1.02  *P =* 0.033  CD8+ Cell Number (high):  HR = 0.99  95% Cl = 0.97-1.01  P = 0.181  CD3+ Cell Number (high):  HR = 0.99  95% Cl = 0.98-1.00  *P = 0.142*  CD4+ Cell Number (high):  HR = 0.98  95% Cl = 0.96-1.00  P = 0.082  CD20+ Cell Number (high):  HR = 0.98  95% Cl = 0.96-1.00  P = 0.186  FoxP3 Cell Number (High):  HR = 0.98  95% Cl = 0.93-1.03  P = 0.374  PD-L1 Expression (High):  HR = 0.32  95% Cl = 0.11-0.94  P = 0.039 | Univariate analysis:  PD-1 Cell number (high):  HR = 0.94  95% Cl = 0.85-1.04  *P =* 0.213  miR-197 expression (high):  HR = 1.01  95% Cl = 1.00-1.02  *P =* 0.089  CD8+ Cell Number (high):  HR = 0.99  95% Cl = 0.96-1.01  P = 0.282  CD3+ Cell Number (high):  HR = 1.00  95% Cl = 0.98-1.01  *P = 0.552*  CD4+ Cell Number (high):  HR = 0.98  95% Cl = 0.96-1.01  P = 0.187  CD20+ Cell Number (high):  HR = 0.99  95% Cl = 0.97-1.02  P = 0.496  FoxP3 Cell Number (High):  HR = 1.01  95% Cl = 0.96-1.06  P = 0.754  PD-L1 Expression (High):  HR = 0.25  95% Cl = 0.06-1.12  P = 0.070 | ? | ? | IHC | PD-1  MiR-197  CD8  CD3  CD4  CD20  FoXP3  PD-L1 | ? | PubMed |
| **Takahashi et al., 2017** | Cancer-associated fibroblasts promote an immunosuppressive microenvironment through the induction and accumulation of protumoural macrophages | The infiltration of CAF was associated with the numbers of CD68-positive and CD163-positive macrophages  Correlated with lymphatic invasion, vascular invasion, lymph node involvement and TNM stage  Infiltration of CAFs was identified as an independent prognostic factor in OSCC | Mean±SD CD68+:  204±200  Mean±SD CD163+:  64±55 | CAFs play pivotal roles in shaping the tumour immunosuppressive microenvironment in OSCC, and therapeutic strategies to reverse the CAF-mediated immunosuppressive microenvironment need to be considered in order to increase the effectiveness of conventional therapies as well as immunotherapies. | 2017 | Japan | 73 | 36-92 | ? | ? | Oral squamous cell carcinoma | TNM stage:  I-IV  T stage:  T1-T4 | Tumour stroma | Univariate analysis:  CD68:  HR = 2.332  95% Cl = 0.859-6.332  P = 0.10  CD163:  HR = 2.335  95% Cl = 0.993 - 5.490  P = 0.05  Multivariate analysis:  CD163:  HR = 1.114  95% Cl = 0.345 - 3.597  P = 0.86 | ? | Univariate analysis:  CD68:  HR = 4.307  95% Cl = 1.259-14.742  P = 0.02  CD163:  HR = 2.304  95% Cl = 0.957 - 5.543  P = 0.06  Multivariate analysis:  CD68:  HR = 2.382  95% Cl = 0.633 - 8.961  P = 0.20  CD163:  HR = 1.322  95% Cl = 0.524-3.338  P = 0.55 | ? | IHC | CD68  CD163 | ? | PubMed |
| **Matsuoka et al., 2015** | The tumour stromal features are associated with resistance to 5‐FU‐based chemoradiotherapy and a poor prognosis in patients with oral squamous cell carcinoma | The group with high level of CAFs revealed an incidence of advanced pT- and pN-stage cases was significantly higher than that in the group with the low level  A high TAMs tumour expression was significantly correlated with a poor response to preoperative chemoradiotherapy  A Kaplan–Meier analysis revealed that higher numbers of CAFs and TAMs were significantly correlated with a poor prognosis | Median number:  CD163+ = 3.2/HPFs, range 0.0-9.0 | High TAM expression level contributed to resistance to chemoradiotherapy and a poor prognosis in patients with OSCC undergoing 5-FU-based to chemoradiotherapy  High CAF expression is associated with an increased p-T stage and p-N stage of clinical OSCC and poor prognosis  Our present findings suggest that the status of the CAFs and TAMs may be appreciable to making treatment decisions to improve the survival of OSCC patients.  Additional studies to clarify the functional roles of the tumour microenvironment in the resistance of cancer cells to chemo- and/or radiotherapy and the therapeutic efficacy of targeting TAMs need to be performed to confirm the clinical significance of our findings. | 60 | Japan | ? | 33-87 | ? | ? | Oral squamous cell carcinoma | TNM stage: I-IV | Tumour stroma at invasive front | Multivariate analysis:  CD163:  HR = 2.30  95% Cl = 0.65-8.10 | ? | ? | ? | IHC | CD163 | ? | PubMed |
| **Kikuchi et al., 2021** | Clinical significance of tumour‐associated immune cells in patients with oral squamous cell carcinoma | Multivariate analysis results adjusted by the pathological stage, resection margin, and extracapsular extension showed that a high number of PD-L1 + TAICs and intratumoural CD68+ TAICs were independent positive and negative prognostic markers | CPS cut-off values of ≥1 and ≥20, the prevalence of PD-L1 expression was 85% (88/103, 85%) and 15% (15/103, 15%)  Median Number:  PD-L1 = 10  CD68+ = 50 | PD-L1+ TAICs in the tumour microenvironment and CD68+ TAICs in the intratumoural area could act as novel biomarkers for predicting overall survival outcomes in OSCC patients. | 2021 | Japan | 103 | 30-92 | 60 | 43 | Oral squamous cell carcinoma | TNM stage: I-IV | Tumour stroma, intra-tumoural compartment | Univariate analysis:  CD68:  HR = 0.84  95% Cl = 0.31-2.26  PD-L1: | ? | ? | ? | IHC | CD68  PD-L1 | ? | PubMed |
| **Lenouvel et al., 2021** | Clinicopathological and prognostic significance of PD‐L1 in oral cancer: A preliminary retrospective immunohistochemistry study | Positive PD-L1 staining was found in 58% of tumours and was significantly more likely in non-smokers, non-drinkers and in tongue squamous cell carcinomas  Increased PD-L1 was also associated with increased lymphocyte infiltration as well as PD-L1 staining in lymphocytes and the epithelium adjacent to tumour invasion.  No survival benefit was seen from PD-L1 expression in tumour cells | - 32 (58%) expressed PD-L1 at a 5% cut-off | PD-L1 expression is more common in non-smokers and non-drinkers, and its presence in the adjacent non-tumour epithelium suggests it may be involved in early oncogenesis.  We have also shown PD-L1 expression to be significantly associated with tongue OSCC, although further research is needed to confirm this association. | 2021 | Spain | 55 | 42-87 | ? | ? | Oral squamous cell carcinoma | TNM stage: I-IV | ? | Univariate analysis:  PD-L1:  HR= 0.58  95% Cl = 0.14-2.45 | ? | ? | ? | IHC | PD-L1 | ? | PubMed |

**Supplementary table 2:** Assessment criteria to evaluate the quality of included studies in current meta-analysis according to the REMARK guidelines.

| **Checklist Items** | **Criteria** |
| --- | --- |
| ***1. Samples*** | Group with a well-defined study population. Patients that have received therapy must be known. Authors inform if all patients receive the same treatment or not. |
| ***2. Clinical data of the group*** | The basic clinical data such as number of patients, age, gender, tumour stage (clinical stage and histopathologic grade provided) |
| ***3. Immunohistochemistry*** | Straightforward, detailed and easy to follow staining protocol. |
| ***4. Prognostication*** | Clearly defined outcome in regard to duration of follow-up.  Analysed survival endpoints were defined (e.g. overall survival (OS))  Direction of effect on survival analysis in Kaplan-Meier curves. |
| ***5. Statistics*** | Cutoff point, which used to subgroup the cases into high or low risk groups was informed.  Estimated values (HR, 95% Cl and P-value) describing the assessed biomarker and outcome was provided including either univariate or multivariate analyses.  Statistical analysis (e.g., Cox’s proportional hazards regression model) was performed to adjust the biomarker effect for investigated prognostic factors. |
| ***6. Prognostic Factors*** | The prognostic value of the prognostic factors was included  The relationship between the evaluated biomarker or biomarkers and known prognostic factors were reported. |

**Supplementary table 3:** Data extraction of included studies investigating the CD163, CD68 and PD-L1 in OSCC patients

| **Author/year** | **Biomarker** | **Follow-up (months)** | **Cut-off point** | **Univariate or multivariate analysis** | **Overall Survival (HR (Hazard ratio), 95% Cl)** | **P Value (*P*)** |
| --- | --- | --- | --- | --- | --- | --- |
| ***Fujii et al., 2012*** | CD163 | N/A | Median, 1.6 HPF (high pass filter) (CD163) | Multivariate | 2.64, 1.02-6.80 | 0.045 |
| ***Fujita et al., 2014*** | CD163 | N/A | Median | Multivariate | 4.53, 0.75-27.36 (Estimated) | 0.006 |
| ***Wang et al., 2014*** | CD163 | 61.5 (Median) | Median | Multivariate | 3.56, 1.67-7.59 | 0.001 |
| ***Matsuoka et al., 2015*** | CD163 | N/A | Median, 3.2 HPF (CD163) | Multivariate | 2.30, 0.65-8.10 | 0.195 |
| ***Takahashi et al., 2017*** | CD68, CD163 | 30.5 (Median) | Median, 204  ± 200 (CD68), 64 ± 55 (CD163) | Univariate (CD68)  Multivariate (CD163) | 1.11. 0.34-3.70 (CD163)  2.33, 1.00-5.45 (CD68) | 0.86 (CD163)  0.10 (CD68) |
| ***Ni et al., 2015*** | CD68 | N/A | ≥75% | Univariate | 1.39, 0.28-6.89 | 0.846 |
| ***Fang et al., 2017*** | CD68 | 48 (Median) | Mean | Multivariate | 0.73, 0.43-1.31 | 0.293 |
| ***Kikuchi et al., 2021*** | CD68, PD-L1 | 40.8 (Median) | Median,  ≥1 and ≥20 | Univariate (CD68)  Univariate (PD-L1) | 0.84, 0.31-2.26 (CD68)  0.50, 0.18-1.39 (PD-L1) | 0.73 (CD68)  0.19 (PD-L1) |
| ***Lin et al., 2015*** | PD-L1 | 45.6 (Mean) | N/A | Univariate | 1.21, 0.89-1.64 | 0.225 |
| ***Kogashiwa et al., 2017*** | PD-L1 | 40.6 (Mean) | Mean | Multivariate | 0.26, 0.10-0.65 | 0.008 |
| ***Ahn et al., 2017*** | PD-L1 | 44.3 (Mean) | N/A | Univariate | 0.32, 0.11-0.93 | 0.039 |
| ***Lenouvel et al., 2021*** | PD-L1 | 56 (Median) | 5% TPS (tumour proportion score) | Univariate | 0.58, 0.14-2.45 | 0.459 |

**Supplementary figure 1.**

**
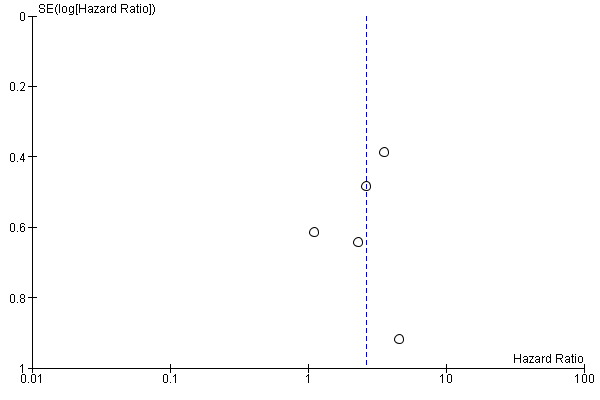
a)**

**b)**


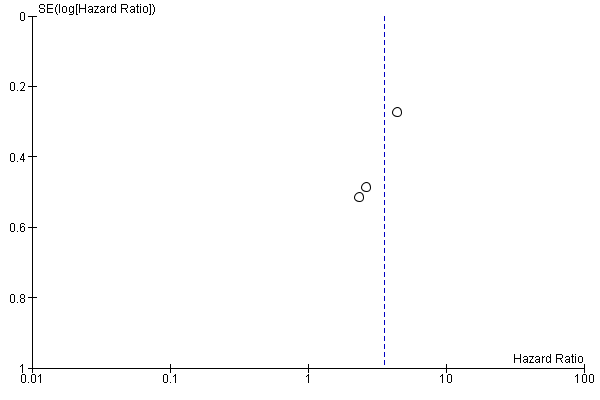


**c)**


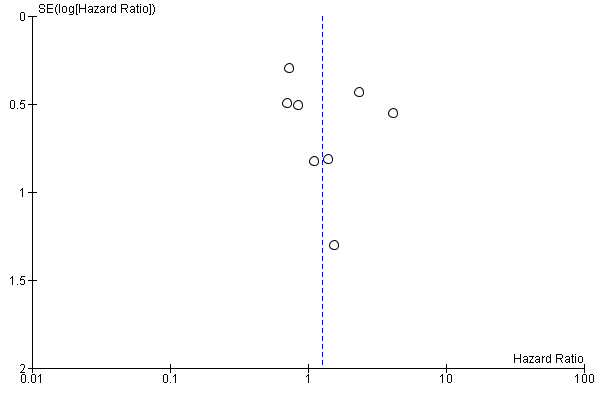


**d)**


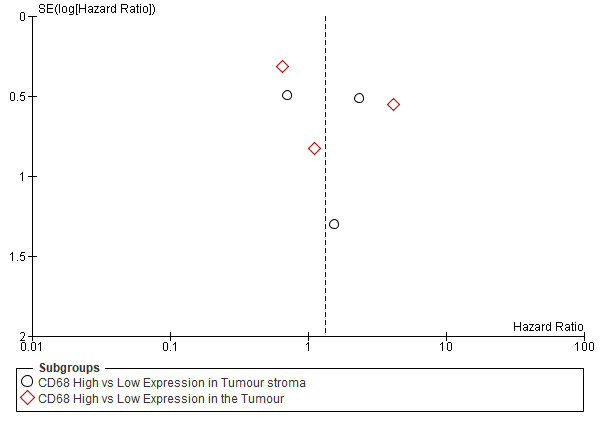


**e)**


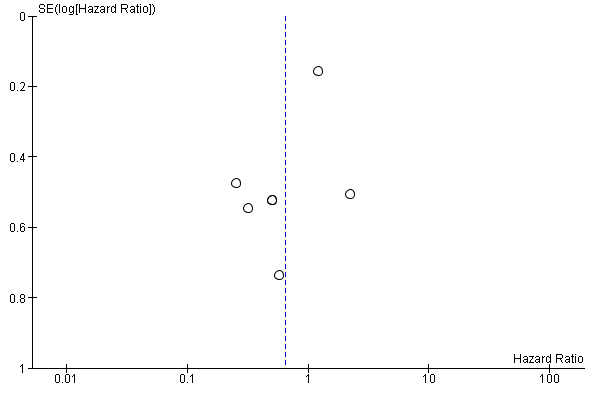


**f)**


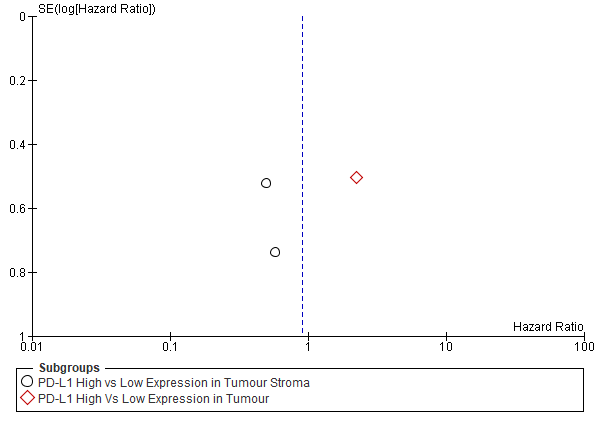


**Supplementary figure 1:** Funnel plot for studies in relation to OSCC prognosis, evaluating publication bias. Standard error of log hazard ratio (y axis) is plotted against its effect size (X axis); where a) CD163^+^ TAMs prognosis in OSCC, b) stromal-located CD163^+^ TAMs prognosis in OSCC, c) CD68^+^ TAMs prognosis in OSCC, d) stroma and intra-tumour localisation of CD68^+^ TAMs prognosis in OSCC, e) PD-L1 prognosis in OSCC and f) stroma and intra-tumour localisation of PD-L1 prognosis in OSCC.
